# General improvements versus interruptive or non-interruptive alerts in the blood order set: study protocol for a randomized control trial to improve packed red blood cell utilization

**DOI:** 10.1186/s13063-023-07319-8

**Published:** 2023-05-08

**Authors:** Neelam Mistry, Vanessa Richardson, Evan Carey, Samuel Porter, Sharon Pincus, Sylvie Novins-Montague, Megan Elmer, Chen-Tan Lin, P. Michael Ho, Tyler Anstett

**Affiliations:** 1 Division of Hospital Medicine, Department of Medicine, University of Colorado Anschutz Medical Campus, 12401 East 17Th Avenue, Mailstop F-782, Aurora, CO 80045 USA; 2 Department of Biostatistics and Informatics, Colorado School of Public Health, University of Colorado Anschutz Medical Campus, Aurora, CO USA; 3 The NavLab, an Adult and Child Consortium of Outcome Research and Delivery Science (ACCORDS) Program, University of Colorado School of Medicine, Aurora, CO USA; 4 Division of Internal Medicine, Department of Medicine, University of Colorado Anschutz Medical Campus, Aurora, CO USA; 5 Division of Cardiology, Department of Medicine, University of Colorado Anschutz Medical Campus, Aurora, CO USA

**Keywords:** Blood transfusions, Clinical decision support, Evidence-based transfusion, Transfusion threshold, Packed red blood cells, Randomized control trial

## Abstract

**Background:**

Blood transfusions can serve as a life-saving treatment, but inappropriate blood product transfusions can result in patient harm and excess costs for health systems. Despite published evidence supporting restricted packed red blood cell (pRBC) usage, many providers transfuse outside of guidelines. Here, we report a novel prospective, randomized control trial to increase guideline-concordant pRBC transfusions comparing three variations of clinical decision support (CDS) in the electronic health record (EHR).

**Methods:**

All inpatient providers at University of Colorado Hospital (UCH) who order blood transfusions were randomized in a 1:1:1 fashion to the three arms of the study: (1) general order set improvements, (2) general order set improvements plus non-interruptive in-line help text alert, and (3) general order set improvements plus interruptive alert. Transfusing providers received the same randomized order set changes for 18 months. The primary outcome of this study is the guideline-concordant rate of pRBC transfusions. The primary objective of this study is to compare the group using the new interface (arm 1) versus the two groups using the new interface with interruptive or non-interruptive alerts (arms 2 and 3, combined). The secondary objectives compare guideline-concordant transfusion rates between arm 2 and arm 3 as well as comparing all of arms of the study in aggregate to historical controls. This trial concluded after 12 months on April 5, 2022.

**Discussion:**

CDS tools can increase guideline-concordant behavior. This trial will examine three different CDS tools to determine which type is most effective at increasing guideline-concordant blood transfusions.

**Trial registration:**

Registered on ClinicalTrials.gov 3/20/21, NCT04823273. Approved by University of Colorado Institutional Review Board (19–0918), protocol version 1 4/19/2019, approved 4/30/2019.

## Introduction

### Background

While blood transfusions can serve as a life-saving treatment, inappropriate blood product transfusions can result in patient harm and excess costs for health systems [1]. There is a growing body of evidence that supports a restrictive transfusion strategy [2–6]. With a few specific exceptions, the American Association of Blood Banks (AABB) recommends limiting packed red blood cell (pRBC) transfusions to patients with a hemoglobin level less than 7 g/dL for adult hospitalized patients, including critically ill patients [7]. Despite established guidelines, providers frequently transfuse for inappropriate hemoglobin values 22–93.5% of the time [8–10].

Clinical decision support (CDS) embedded into the electronic health record (EHR) can change provider behavior, namely improving process and outcome measures across multiple healthcare settings [11–15]. Goodnough et al. implemented single-center CDS to improve blood utilization which consisted of an interruptive alert that provided a link to relevant literature and an acknowledgement reason for the transfusion. This intervention led to a statistically significant improvement in guideline-concordant blood transfusions [16]. Similar outcomes were replicated by Jenkins et al. in a prospective trial single-center initiative using an interruptive alert, other EHR changes, plus an educational campaign to reduce unnecessary transfusions and costs [17]. While both studies demonstrated the significant positive effects of CDS tools on blood transfusions, information on which specific intervention of those implemented led to these results is unknown. Further, both studies tested the use of interruptive alerts. It is not known whether other types of CDS may be as or more effective in changing provider ordering of pRBCs.

Randomized control trials (RCTs) with CDS tools across various quality and safety measures have been implemented within EHRs to nudge providers toward evidence-based care [18, 19]. There is evidence supporting computerized physician order entry (CPOE) based CDS interventions in general, but there is little published regarding the specific types of CDS that are most efficient in generating a change in behavior. As such, we developed a pragmatic, prospective randomized trial to evaluate the effectiveness of specific CDS elements on transfusion practices of providers in a large academic medical center. Specifically, we sought to identify whether alerts and how they are displayed (interruptive or non-interruptive) affect provider ordering behavior. This study randomized providers to three different CDS tools to identify the relative effectiveness of each specific CDS. To our knowledge, there are no RCTs to date that randomize providers to different CDS interventions to increase guideline-concordant blood product ordering behaviors. Here, we report the methods employed in the study.

## Methods and study design

### Study aim and settings

This is a single-center, randomized control trial conducted at University of Colorado Hospital (UCH), a level 1 trauma center and primary teaching hospital for the University of Colorado School of Medicine, located in Aurora, Colorado, part of a larger UCHealth health system. UCH has 678 beds and is a transplant center for heart, kidney, pancreas, liver, and lung transplants.

### Eligibility criteria

All providers (physicians, physician assistants, nurse practitioners, nurses, and trainees) who worked at UCH during the study period with ordering privileges through the EHR were randomized to one of three study arms using 1:1:1 randomization to each of the study arms.

### Study objective and outcomes

The objective of this study is to identify if a specific CDS—general improvements, general improvements plus non-interruptive alerts, or general improvements plus interruptive alerts—is more effective at increasing the proportion of guideline-concordant pRBC transfusions. Ordering providers were randomized to a new ordering interface with general improvements (arm 1), a new ordering interface with non-interruptive alerts (arm 2), or a new ordering interface with interruptive alerts (arm 3). The primary objective compares the proportion of guideline-concordant pRBC transfusions between the general improvement orders interface (arm 1) versus the interfaces with alerts (arms 2 and 3, combined). Accordingly, the primary outcome is the proportion of guideline-concordant pRBC transfusions based on the patient’s most proximal pre-order hemoglobin value and the number of units ordered with guideline concordance as described below. The secondary objective compares the proportion of guideline-concordant pRBC transfusions between the non-interruptive alert order set (arm 2) versus the interruptive alerts order set (arm 3). Exploratory objectives will compare historical pRBC transfusions data (preintervention interface) to arms 1–3 combined. We hypothesize more guideline-concordant pRBC transfusions in the combined alert arms (arms 2 and 3) than the general improvements arm (arm 1).

**Fig. 1:**
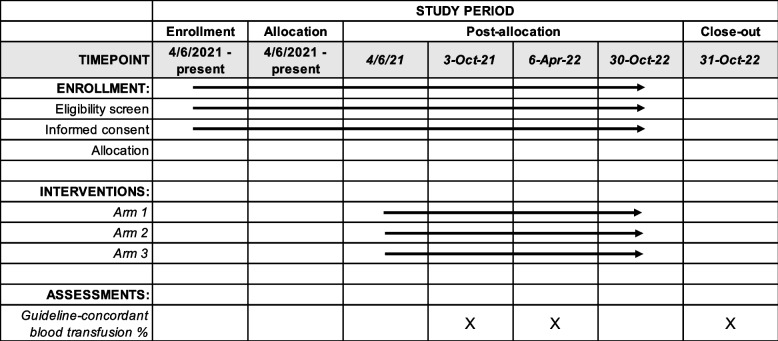
Planned study period schedule with rolling provider enrollment

The Standard Protocol Items: Recommendations for Interventional Trials (SPIRIT) reporting guidelines were used [20]. Fig. 1outlines schedule of enrollment, interventions, and assessments [20].

Funding for data analytics personnel support for this project was provided by the NavLab which was funded by a University of Colorado School of Medicine Dean’s Transformational Research Grant. The sponsor played no part in study design, collection, management, analysis, and interpretation of data, writing of the report, and the decision to submit the report for publication.

### Trial design and randomization

The initial user group of existing providers (*n* = 1640) were randomized 1:1:1 to the three study arms on April 6, 2021. Each time the ordering provider subsequently placed orders to prepare or transfuse pRBC, the provider remained in the same arm as per the initial randomization. In the baseline data examined, the number of pRBC transfusion events ordered was highly variable between providers. To balance providers with high and low ordering behavior, providers were randomized in blocks based on their ordering behavior at baseline. The average monthly ordering rate for each provider was calculated and the distribution of average monthly ordering rates divided into groups by quartile. Providers were then block randomized according to their average monthly ordering rate quartile group. All eligible providers who started at UCH after the initial randomization were similarly allocated 1:1:1 to one of the three study arms. This study was classified as quality improvement and thus non-human subject research so did not require informed consent from providers to enroll in the intervention arms per the study site’s institutional review board (IRB). No identifying images or other personal or clinical details of providers or patients are included here or will be presented in reports of the trial results. This trial does not involve collecting biological specimens for storage. There is no anticipated harm or compensation for trial participation. The project management group is composed of all authors listed in this trial. A subset of the authors met regularly to review trial progress and were responsible for monitoring trial results every 6 months to evaluate outcomes data. An interim analysis was conducted every 6 months to implement a-priori specified stopping criteria, specifically a 20% effect size as described below. The authors met with EHR analysts to implement the order set changes before the start of the trial. Day-to-day EHR support for providers is provided through the EHR help desk. EHR help desk personnel could reach out to the principal investigator for questions. This intervention was considered low-risk, and therefore, a data monitoring committee was not considered.

### Treatment conditions, participant population, and study setting

Our study population included providers working at University of Colorado Hospital (UCH), who cared for patients who are admitted to UCH, who are 18 years of age or older, and order pRBC transfusions on inpatient medical, surgical, labor and delivery units, or in the emergency department at the hospital. We excluded transfusions performed in operating rooms or procedural areas as well as transfusions performed as part of a massive transfusion protocol (MTP). There was no patient or public involvement in the design of this study protocol.

Treatment conditions include anemia of any cause where a provider felt a pRBC blood transfusion is clinically indicated.

Providers were randomized to one of three arms if they were a physician (MD), physician assistant (PA), nurse practitioner (NP), registered nurse (RN), or trainees in any of these licensed training programs who can order pRBCs in the EHR, Epic Systems Corporation, at UCH. All providers were prospectively randomized into a study arm; the providers continued clinical care as usual but encountered their assigned study intervention arm automatically via the EHR if ordering a blood transfusion outside of guideline recommended thresholds. There were no special criteria for modifying a provider’s allocated intervention during the trial. The allocated intervention would be discontinued if the provider left the hospital where the study was conducted.

### Intervention and implementation strategies

**Fig. 2:**
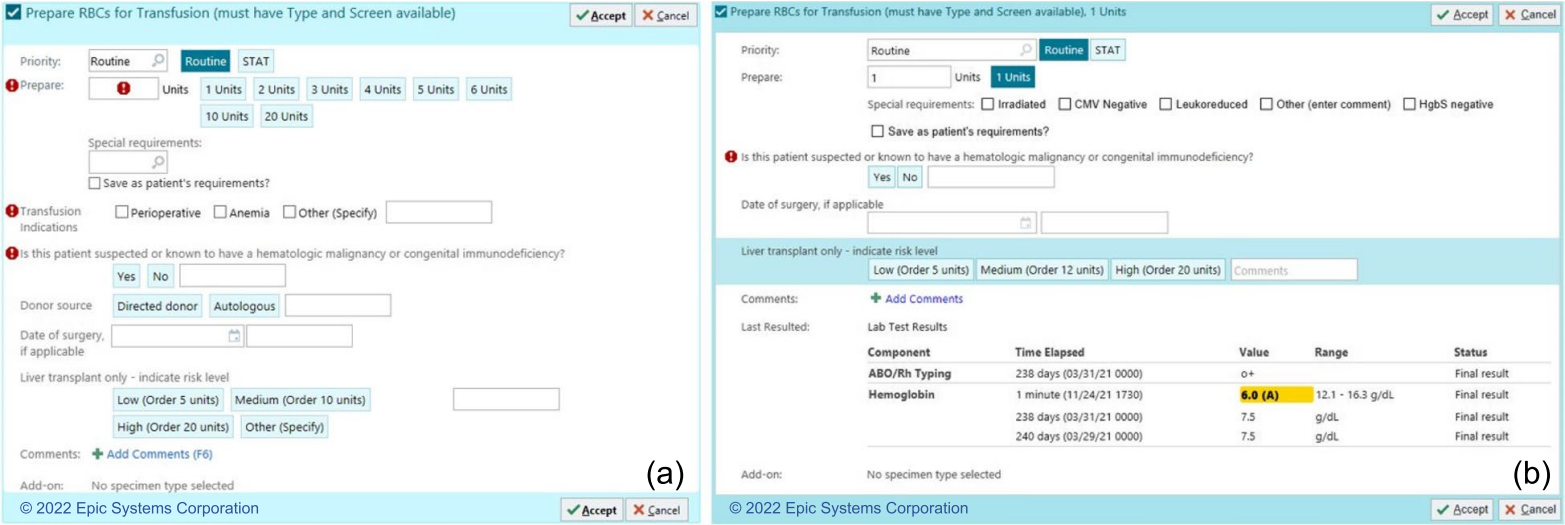
**a** Pre-intervention Prepare pRBC order. **b** Post-intervention general improvement changes made for all users to the Prepare order

**Fig. 3:**
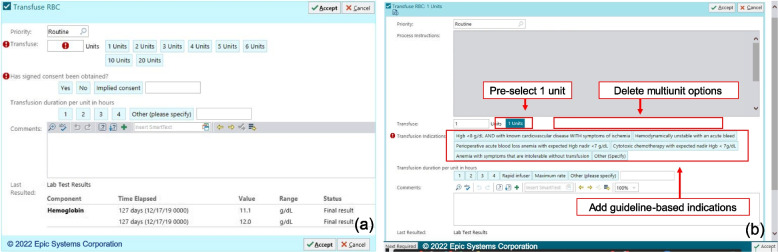
**a** Pre-intervention pRBC Transfuse order. **b** Post-intervention general improvement changes made for all users to the Transfuse order

Prior to this intervention, all providers received the same prepare and transfuse pRBC blood transfusion orders as noted in Fig. 2a and Fig. 3a, respectively. At baseline, both the Prepare and Transfuse orders had multiple button options for number of units to prepare or transfuse. The Prepare order notably required a nonspecific indication for transfusion, with options of “Perioperative,” “Anemia,” or “Other (specify)”.

The following interventions—changes in the blood transfusion order sets—were implemented in the three arms of this study in April 2021. This trial is now concluded.

**Fig. 4:**
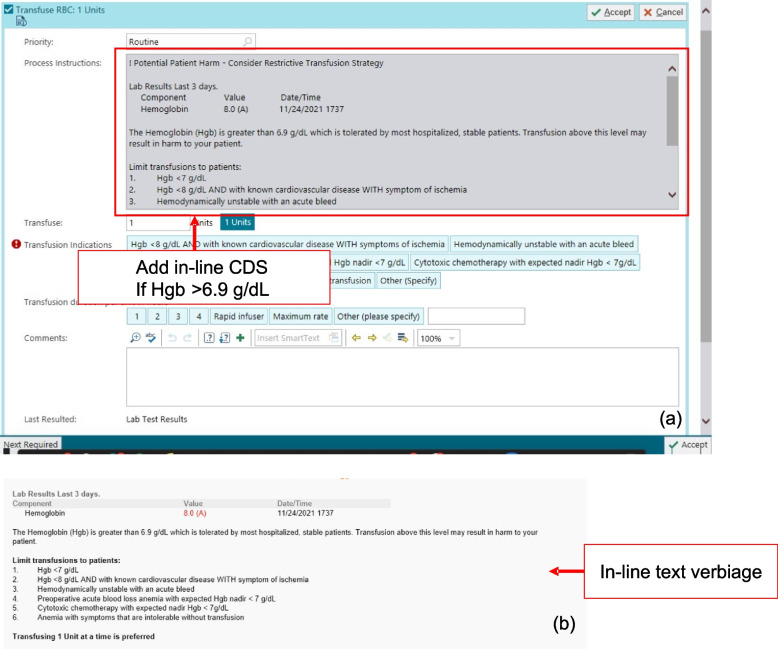
**a** Non-interruptive in-line CDS (arm 2) if hemoglobin (HGB)  > 6.9 g/dL. **b** Text verbiage of non-interruptive in-line CDS

**Fig. 5:**
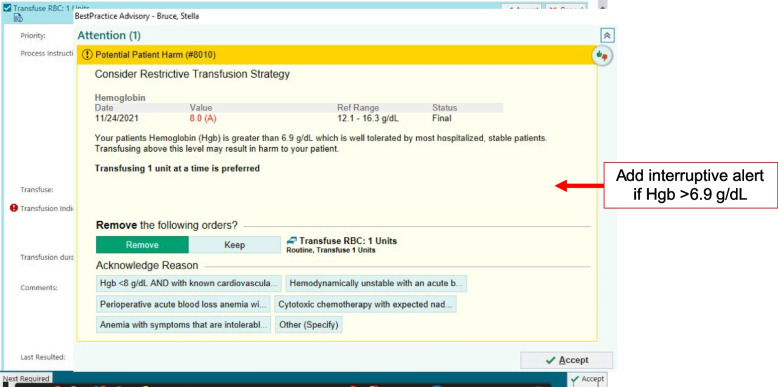
Interruptive CDS alert (arm 3) if HGB > 6.9 g/dL

1) Arm 1—General improvements (no alerts): Using the expertise of the study personnel, EHR system architects, and incorporating feedback from the users who participated in structured user-centered design sessions, the blood transfusion order-set as well as the individual Prepare and Transfuse orders were changed (Fig. 2b and Fig. 3b, respectively). Changes to the Prepare order included removing the indications and the multi-unit prepare buttons. Changes to the Transfuse order included removing the multi-unit transfusion buttons and adding guideline-concordant exceptions to the recommended transfusion threshold of a hemoglobin less than 7.0 g/dL. These changes reflect behavioral nudge principles to guide users to transfuse according to guidelines. A behavioral nudge is a small change in framing choice that alters people’s behavior in a predictable way [21]. These changes were intended to be more intuitive for ordering clinicians and to reduce the number of clicks, decisions, and overall cognitive load. Additionally, by automatically choosing one unit to transfuse, eliminating readily available options for multi-unit transfusions (adding an extra step to transfuse more than one unit at a time), and forcing providers to choose from a list of guideline-concordant indications, these changes incorporate behavioral nudges to encourage providers to order within guidelines.

2) Arm 2—Non-interruptive alert (in-line help text): In addition to the changes in the general improvement arm, providers randomized to the in-line help text arm were shown a non-interruptive text alert detailing evidence-based transfusion recommendations that appeared if the most recent hemoglobin level was above 6.9 g/dL (Fig. 4). This text appeared within the transfusion order and order-set but was non-interruptive as it did not require users to acknowledge the text nor does it require any additional keystrokes or clicks.

3) Arm 3—Interruptive alert: In addition to the changes in the general improvement arm, providers randomized to the interruptive alert arm were shown an interruptive text alert detailing evidence-based transfusion recommendations that appear if the most recent hemoglobin level is above 6.9 g/dL (Fig. 5). In contrast to the in-line help text arm, this arm included an interruptive “pop-up” alert that appeared when the user selects the “Transfuse Order.” This alert offered users the option to remove the order which results in no-blood product ordered. Alternatively, users may continue to order blood and are asked to select the reason for proceeding with the intended order with the selections reflective of the guideline indications for blood transfusions.

It is important to note that in each of the arms, the alerts (non-interruptive and interruptive) as well as the required selection of indications for transfusion were only displayed if the patient’s most recent hemoglobin value was above 6.9 g/dL. Providers ordering pRBCs for patients with a hemoglobin less 7.0 g/dL were not required to select an indication for transfusion nor were they shown any alerts. The intention of only showing the alerts and requiring providers to choose an indication when the hemoglobin is above 6.9 g/dL is to allow providers practicing within guidelines to proceed without extra work. Further, we hypothesize that if providers only see certain elements when their behavior might be guideline discordant, this will serve to alert them to consider a different action. Making it more cumbersome to order and showing alerts when placing orders outside of guidelines follows nudge principles. If a patient did not have a hemoglobin level in the system within seven days of the order, the interventions behaved as if the hemoglobin was greater than 6.9 g/dL. This was an EHR intervention with nudge approaches, but providers always had the opportunity to bypass the alerts and order blood as clinically indicated. These interventions did not prevent providers from continuing forward with their original clinical decision making. Therefore, we do not anticipate any adverse events (AE) or harms from the intervention. If a provider felt there was an adverse event or error related to this trial, the provider could report this issue through established, hospital-wide patient safety tracking system indicating expectedness, seriousness, severity, and causality.

No part of this trial was blinded. However, the outcome of guideline-concordant pRBC transfusion was objectively defined prior to implementing the trial and based on data directly available from the EHR (most recent pre-order hemoglobin and the number of units ordered). Thus, the outcome of guideline-concordant pRBC is assessed as part of usual clinical care and available in the EHR. The data analysts used the most recent pre-order hemoglobin and the number of units ordered and then implemented the algorithm to determine guideline-concordant pRBC transfusion. This algorithm was implemented identically across all study arms.

At the start of the study, 1640 existing providers were randomized in a 1:1:1 fashion to each of the arms by study personnel. All eligible providers who started at UCH after the initial randomization were allocated to one of the three study arms. Data was collected via EHR data extraction and stored in a secure database to prevent breach of protected health information and personal identifying information for a provider will be removed.

## Measures and analysis plan

### Planned analysis

#### Descriptive analyses

Provider traits and patient traits stratified by study arm assignment will be summarized with descriptive statistics to check for balance across study arms. Imbalance will be summarized as standardized mean differences.

#### Description of outcome (guideline-concordant pRBC transfusion)

The binary outcome of interest, guideline concordance of pRBC ordering, is defined based on the patient’s most recent pre-order hemoglobin value and the number of units ordered. An order is guideline concordant if the pre-order hemoglobin value is less than 7.0 g/dL and one unit is ordered or if the pre-order hemoglobin value is less than 6.0 g/dL and two units are ordered. Because there are other indications for pRBC ordering beyond a pre-order hemoglobin value, any order not meeting guideline concordance as described was labeled as potentially guideline discordant. We planned to collect outcome data for 18 months following the start date of the study.

#### Treatment comparisons

The a priori primary objective comparison is between the group using the new interface (arm 1) and the two groups using the new interface with non-interruptive or interruptive alerts combined (arms 2 and 3, respectively).

If the a priori primary objective comparison is statistically significant, we will further test the secondary comparison for differences in compliance between the different alert groups, arm 2 versus arm 3.

We will conduct an exploratory comparison of the historical pre-trial data to the post trial onset data. If the primary objective comparison is statistically significant, we will compare the pre-trial data to arm 1 and then 2 and 3 combined. If the primary objective comparison is not statistically significant (no difference across arms), we will compare the pre-trial data to arms 1, 2, and 3 combined.

Hypothesis testing for both primary and secondary comparisons of the trial data will be implemented using generalized linear mixed models with the binary outcome of guideline-concordant order, group comparison(s) as a fixed effect, and a random intercept for provider. Significance testing will be set at *p* < 0.05.

The comparison of historical pre-trial data versus the post-trial onset data will be implemented using generalized linear mixed models with the binary outcome of guideline-concordant order (yes/no). We will implement an interrupted time series analysis by parameterizing this model with fixed effects including an intercept, a term for post-trial onset (intercept shift), a linear time trend, and an interaction between post-trial onset and linear time trend (slope difference). Pre-trial data is available for two years prior to the trial onset. The COVID pandemic likely affected the outcome of interest. If there are notable effects on the pre-trial (but post COVID onset) trend in guideline-concordant ordering, we will exclude the immediate period post-COVID from the historical pre-trial trend estimation.

Interim analyses were conducted at 6 and 12 months to test the primary objective comparison. If a statistically significant result of a 20% reduction in potentially guideline discordant behavior with a significance level of 0.05, the trial would have been stopped early and we would have declared one arm superior to the other if needed—thus, we set the bar high (at least 20% effect size). For example, if the probability of potentially guideline discordant behavior was 0.5 in one group, a difference of 0.2 × 0.5 = 0.1 is the effect size. Study personnel noted in the author list (EC, VR, MH) have access to the interim results and base trial termination on these analyses.

All providers with pRBC ordering privileges at the start of the trial were prospectively enrolled in the study. There were no specific plans to promote participant retention as participation in this trial is dependent only on clinical privileges at the trial center. All providers enrolled were considered 100% adherent to the study protocol as they receive their designated EHR intervention arm each time they order blood products outside of guidelines. If a provider left UCH, their data contribution was included in the study up until the time of their departure from the system.

### Statistical power

Simulations were conducted to estimate power and sample size for this study. In all simulations, providers were randomly assigned to the three different treatment groups with equal probability. Since all providers in the system were enrolled into an arm, there were no possible strategies to increase participation enrollment beyond running the trial for a longer period of time, as all new providers during the study period were enrolled into an arm. The following assumptions were made about the data generating process: (1) the average number of transfusion orders across UCH was approximately 30 per day at baseline, (2) opportunities to order a blood transfusion occurred at different rates for different providers, (3) providers encountered opportunities to order blood at rates of 1 time per day on average for hospitalists and 3 times per day on average for intensivists. In approximately 15% of these opportunities, the patient’s hemoglobin level was less than 7.0 g/dL. At baseline, a provider’s probability of ordering a transfusion was 1 when hemoglobin was less than 7.0 g/dL, 0.2 when hemoglobin was between 7.0 and 8.0, and 0.05 when hemoglobin was greater than 8.0.

We began the simulation with the following assumptions about the data generating process, based on the historical data or on subject matter expert input: (1) there should be on average 30–35 transfusions per day; (2) the number of active providers should be 228, evenly randomized into 3 groups (the total number of providers is not stochastic); (3) there are two different types of providers (high and low frequency): 30% of providers are high-frequency, while 70% of providers are low frequency (this is sampled stochastically in each simulation iteration). High-frequency ordering providers have a daily number of encounters following a Poisson distribution with lambda = 3 (Average of 3 per day). Low-frequency providers have a daily number of encounters following a Poisson distribution with lambda = 1 (average of 1 per day).

Patient hemoglobin (HGB) values were next simulated for all encounters, assuming HGB is normally distributed with mean = 8.3 and SD = 1, truncated at 0. We next simulated transfusion ordering for each encounter, conditional on the HGB value. The assumed probabilities for transfusion ordering for all groups under the null hypothesis (no difference between arms) is 1 for HGB < 7, 0.2 for 7 <  = HGB < 8, and 0.08 for HGB >  = 8.

We first simulated provider encounters where the provider has the opportunity to order a transfusion. In the observed trial data, we observed transfusions—thus, encounters that do not lead to a transfusion will not be observed. We generated encounters for these providers for time *t* (where *t* is the length of time the trial runs). Then, for each patient encounter, we simulated the HGB value associated with that encounter. We next simulated whether or not an encounter resulted in a transfusion order, which is conditional on HGB value and based on SME feedback. Transfusions are considered potentially guideline discordant if HGB is >  = 7 (any number of units). To explore potential effect sizes of the intervention for the primary hypothesis that groups 2 and 3 have an identical reduction in potentially guideline discordant ordering compared to group 1 (10% reduction, 15% reduction, and 20% reduction), we then decreased the assumed probabilities for transfusion ordering for encounters with HGB >  = 7 by *e*, where *e* is in (0.9,0.85,0.8). Finally, we implemented a logistic regression where each observation is a transfusion order, the outcome is guideline compliance as described above, and a fixed effect for treatment group with 2 levels (group 1 versus groups 2 + 3 combined). We tested the significance of the two-level fixed effect treatment group term against a significance level of 0.05. We replicated this simulation for each potential effect size (10% reduction, 15% reduction, and 20% reduction). To explore the difference in estimated power under each effect size for varying lengths of trial time *t,* we conducted the simulation 2000 times for each potential trial time length *t*, where *t* was 10 weeks up to 50 weeks.

For the primary objective comparison, we assumed that groups 2 and 3 (non-interruptive or interruptive alerts) had an equal probability of guideline-concordant transfusion ordering. Within this scenario, we examined three different effect sizes (the difference in the probability of guideline-concordant transfusion ordering). The first effect size (smallest) assumed a 10% reduction in the probability of a potentially guideline discordant transfusion order occurring. The second effect size assumed a 15% reduction in the probability of a potentially guideline discordant transfusion order occurring. The third effect size (largest) assumed a 20% reduction in the probability of a potentially guideline discordant transfusion order occurring. We conducted a simulation with 2000 replicates for each effect size to estimate the length of time required for the study to achieve 80% power.

Results from these simulations indicate that we will have 80% power to detect an effect size of 15% at 35 weeks and an effect size of 20% at 19 weeks. At 52 weeks, the power to detect an effect size of 10% is 64%. Since there was uncertainty in the expected effect size (no preliminary data available) and the smallest effect size explored would require longer than 52 weeks to achieve 80% power, the trial was designed a-priori to run for 52 weeks.

## Results

The results of this trial will be shared in a separate publication once the study period is complete. Pending results of the study, we plan to implement the most impactful CDS changes at other hospitals in the UCHealth system.

## Discussion

Blood transfusions are a common procedure among hospitalized patients [22]. From 2000 to 2013, the number of hospital inpatient stays with a pRBC increased by 85.8% [23]. Optimal utilization of blood products requires a balance between maximizing patient clinical outcomes while avoiding unnecessary risks associated with pRBC transfusions. There are a myriad of noninfectious adverse outcomes associated with blood transfusions, and while risks may be low (less than 2%), the result of life-threatening adverse reactions can be devastating [24]. The evidence of overutilization indicates that research into changing physician behavior with transfusion practices is imperative to improving utilization within health systems. Avoiding unnecessary transfusions through robust blood management programs is a step in that direction.

While others have described successful implementation of CDS to improve guideline-concordant pRBC transfusions [16, 17], it is not clear which CDS elements of these successful interventions drove the change. Further, previously published studies used interruptive alerts. Though interruptive alerts can provide valuable information and influence behavior, the quantity of alerts in the EHR can lead to provider alert fatigue [25]. If the interventions employed in this trial in the general improvements or non-interruptive alerts improve guideline-concordant behavior without significant differences from an interruptive alert, this can help decrease cognitive load providers face by removing interruptive alerts from the EHR. The primary and secondary objectives will demonstrate how each CDS compares to each other with exploratory objectives to compare to historical controls. This information can demonstrate if specific CDS elements affect decision making to maximize guideline-concordant behaviors while minimizing alerts that do not have benefit.

Further, this study employs nudge theory. As described by Glasgow et al., brief behavioral interventions can influence decision-making and are impactful [21]. Principles of behavioral economics have been incorporated into health interventions to “nudge” people to achieve improved health outcomes [26]. The nudge interventions employed in the general improvements arm along with the alerts in the blood transfusion order set aim to guide provider behavior toward guideline-concordant pRBC transfusion.

Our study has several strengths, including this is a pragmatic, prospective, randomized control trial at a busy academic medical center. All non-procedural inpatient areas are included, covering many subspecialty services, allowing these interventions to be generalizable at other hospitals with an EHR. All guideline concordant exceptions to the transfusion threshold of a hemoglobin of less than 7.0 g/dL causes of anemia are included, and the general improvement changes (arm 1) are applied across arm 2 and arm 3 to capture multiple indications for blood transfusions, which can help target future areas for improvements in blood utilization for specific indications in the future. The pragmatic nature of this trial with randomization allows for generalizability across other institutions that use similar EHRs.

Potential limitations of this study include that this is a single-center trial; however, our institution has robust and rising clinical volumes with a diversity of patients and providers including transplant programs that utilize large volumes of pRBC. Because this study is conducted at a large academic medical center, the frequency of which providers order blood is highly variable; the amount of blood ordered is not consistent among providers, including across trainees, attending physicians, nurses, and advanced practice providers. In addition, despite established guidelines being generally accepted, not all medical specialties have specific transfusion guidelines. To mitigate this, the statistical plan includes a hierarchical model realizing provider level variation, and we blocked the randomization by frequency based on baseline data. Lastly, we can only capture if a provider places an order to transfuse, but not when a provider correctly concludes that they do not need a transfusion and thus do not place an order for transfusion. This limitation is accounted for in the methods and statistical analysis.

## Trial status

This trial is currently closed. The trial was originally intended to run for 12 months; however, the 6-month interim analysis revealed a lower number of transfusions than expected; thus, we elected to extend the trial to 18 months. However, at the next 6 months (week 52–July 2022), we achieved adequate sample size. Since this 12-month interim analysis completed in July 2022 suggested very strongly that there was no statistical significant difference between the arms, the trial was stopped on the original time frame of 52 weeks. All investigators will have access to the final trial dataset. Investigators plan to communicate trial results to the public via journal publication.

## Conclusions

In summary, this randomized control trial aims to dissect specific impacts of CDS on pRBC ordering practices. We hope these changes increase guideline-concordant behaviors with the goal of decreasing unnecessary use of pRBC transfusions, adverse effects, and costs to the health system.

## Data Availability

The datasets generated and/or analyzed during the current study are not publicly available due to the fact that the trial is recently closed and final data is not yet available; once the study has been completed (October 2022), data are available from the corresponding author on reasonable request. Investigators plan to communicate trial results to the public via journal publication.

## Abbreviations

CDS: Clinical decision support
CPOE: Computerized physician order entry
EHR: Electronic health record
MTP: Massive transfusion protocol
pRBC: Packed red blood cells
UCH: University of Colorado Hospital
